# Case Report: Diabetic ketoacidosis after co-administration of empagliflozin and probenecid

**DOI:** 10.12688/wellcomeopenres.19148.2

**Published:** 2024-03-05

**Authors:** William P. Martin, Niamh Reidy, Justin Low, Tomás Ahern

**Affiliations:** 1 Department of Endocrinology, Our Lady of Lourdes Hospital, Drogheda, County Louth, A92 VW28, Ireland; 2 Department of Clinical Microbiology, Our Lady of Lourdes Hospital, Drogheda, County Louth, A92 VW28, Ireland; 3 Department of Infectious Diseases, Our Lady of Lourdes Hospital, Drogheda, County Louth, A92 VW28, Ireland

**Keywords:** diabetic ketoacidosis, DKA, empagliflozin, SGLT2 inhibitor, SGLT2i, probenecid, organic anion transporter 3, OAT3, case report

## Abstract

Sodium-glucose cotransporter-2 (SGLT2) inhibitors are filtered and secreted to their primary site of action in the proximal tubule of the kidney. At this site, SGLT2 inhibitors also reduce renal elimination of ketone bodies, a finding implicated in their propensity to cause ketoacidosis. Many commonly used medications have potential to diminish renal elimination of SGLT2 inhibitors and to compound the effects of SGLT2 inhibitors on renal elimination of ketone bodies by inhibiting tubular secretion of the SGLT2 inhibitor itself and/or ketone bodies. We present a case of severe diabetic ketoacidosis (DKA) in a patient with type 2 diabetes occurring several days after co-prescription of empagliflozin and probenecid. Other than the recent introduction of empagliflozin, no cause for the DKA episode was apparent. A pharmacokinetic interaction between probenecid and empagliflozin, involving organic anion transporter 3 (OAT3), reduces proximal tubular secretion of empagliflozin and increases patient exposure to the drug. Whether or not this phenomenon is sufficient to cause severe DKA is discussed. An alternative explanation as to the DKA aetiology is proposed, wherein probenecid may compound effects of empagliflozin on renal elimination of ketone bodies. We suggest that clinicians exercise caution when prescribing SGLT2 inhibitors alongside pharmacologic inhibitors of, or competitors for, proximal tubular organic anion transporters in patients with diabetes mellitus due to the risk of severe DKA.

## Introduction

Sodium-glucose cotransporter-2 (SGLT2) inhibitors are strongly recommended for patients with type 2 diabetes in the latest clinical practice guidelines based on their demonstrated cardiovascular and renal benefits ^1^. Nevertheless, these medications increase the risk of diabetic ketoacidosis (DKA) ^2, 3^, a severe acute complication of diabetes which carries a substantial risk of morbidity and mortality ^4^.

SGLT2 inhibitors are both filtered and secreted to their primary site of action in the proximal tubule of the kidney ^5^. Although many commonly prescribed medications have the potential to diminish renal elimination of SGLT2 inhibitors by inhibiting their proximal tubular secretion, these interactions are not recognised to increase the risk of DKA nor other side effects of SGLT2 inhibitors and do not influence routine prescription of these medications. We present a case of severe DKA in a patient with type 2 diabetes occurring several days after co-prescription of the SGLT2 inhibitor empagliflozin alongside probenecid, an inhibitor of organic anion transporter 3 (OAT3) ^6^, a transporter which plays a key role in the secretion of empagliflozin into the proximal tubule ^5^. 

## Case report

**Figure 1.**
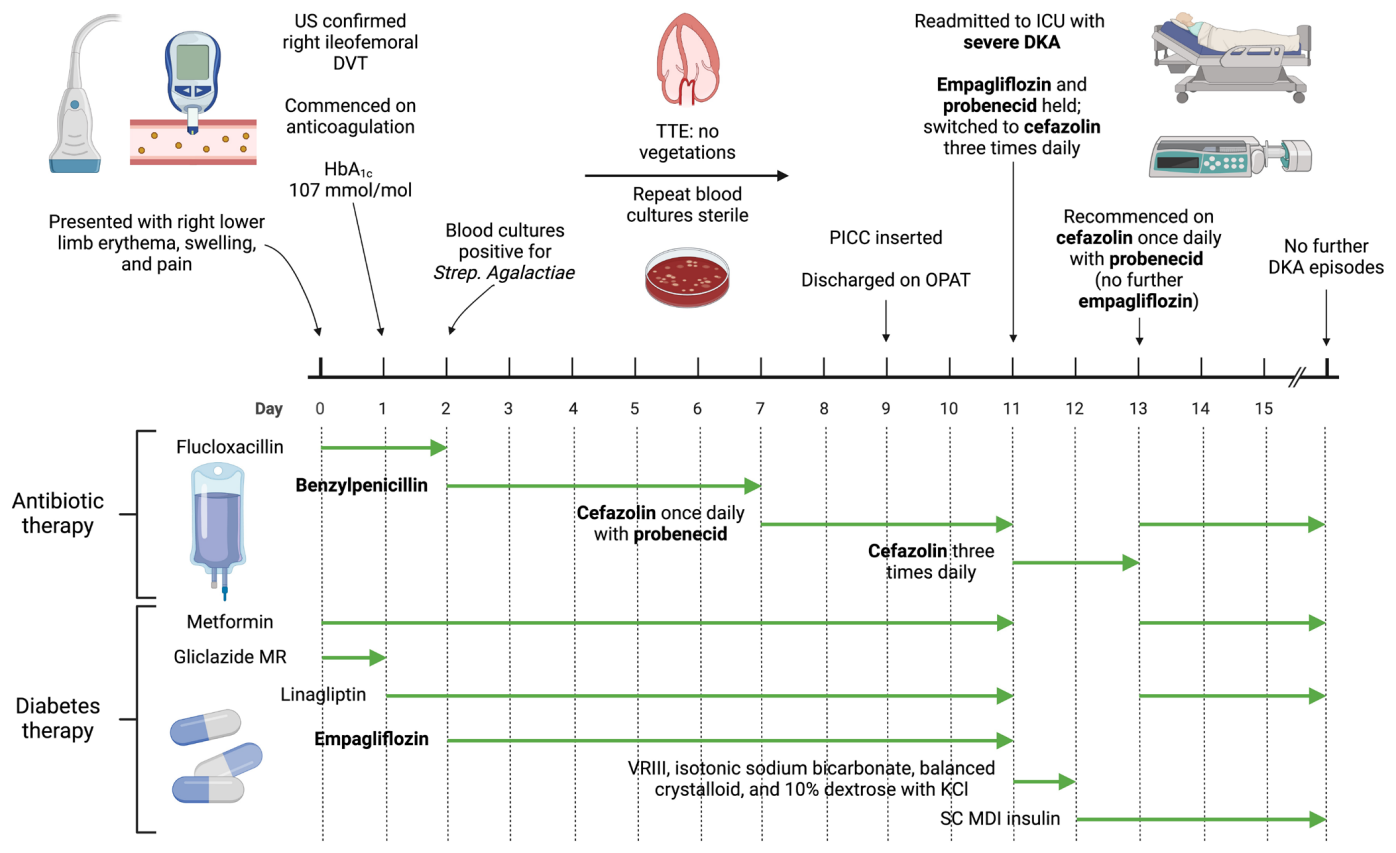
Overview of patient presentation and management, outlining timing of emergence of diabetic ketoacidosis after co-administration of empagliflozin and probenecid. Created with BioRender.com. DKA, diabetic ketoacidosis; DVT, deep vein thrombosis; HbA _1c_, glycated haemoglobin; ICU, intensive care unit; KCl, potassium chloride; MDI, multiple daily injection; MR, modified release; OPAT, outpatient parenteral antibiotic therapy; PICC, peripherally inserted central catheter; SC, subcutaneous; TTE, transthoracic echocardiogram; VRIII, variable rate intravenous insulin infusion. Figure 1 was created with BioRender.com.

A 54-year-old woman with obesity (body-mass index 39.5 kg/m ^2^) and type 2 diabetes mellitus (T2DM, diagnosed 7 years ago) presented to our hospital with a one-week history of right leg redness, swelling, and pain ( Figure 1). There was a family history of type 1 diabetes mellitus (T1DM) diagnosed at 8 years of age in a maternal first cousin and of T2DM diagnosed in a maternal uncle in his mid-50s. The patient lived alone, was independent in activities of daily living, drank alcohol rarely on social occasions, and was a lifelong non-smoker. On examination, the patient had a heart rate of 118 beats per minute (sinus tachycardia on an electrocardiogram), respiratory rate of 19 breaths per minute, blood pressure of 141/91 mmHg, peripheral oxygen saturations of 100% on room air, and a temperature of 38.8 degrees Celsius. Erythema, swelling, and tenderness of the entire right lower limb was noted. Examination of the cardiovascular, respiratory, and abdominal systems was unremarkable. 

Ultrasound imaging confirmed the presence of a right iliofemoral deep vein thrombosis. The blood glycosylated haemoglobin level was 107 mmol/mol. *Streptococcus agalactiae* was cultured in peripheral blood samples. No evidence of infective endocarditis was present on transthoracic echocardiography. The patient was anticoagulated with enoxaparin 1 mg/kg (90 mg) twice daily subcutaneously initially, and later transitioned to apixaban 5 mg twice daily orally. Metformin therapy was continued at a dose of 850 mg three times daily orally, modified release gliclazide 120 mg once daily orally was stopped, and linagliptin 5 mg once daily orally and empagliflozin 10 mg once daily orally were commenced to improve glycaemic control.

The *Streptococcus agalactiae* isolate tested susceptible to penicillin, and treatment with intravenous high dose benzylpenicillin 2.4 g four times daily was commenced with good clinical effect. The source of bacteraemia was unclear; however, given the presence of a deep vein thrombosis, this was presumed to be infected. Blood C-reactive protein and neutrophil count values decreased during her inpatient stay (196 to 33 mg/L and 10.6 to 5.7 x10 ^9^/L, respectively). Prior to discharge, a peripherally inserted central catheter was placed and the antibiotic regimen was switched to intravenous cefazolin (2 g once daily) with oral probenecid (1g once daily) to facilitate a 6-week antibiotic course via the outpatient parenteral antibiotic therapy service.

The patient was readmitted 48 hours later with severe DKA. The patient’s infection was well controlled at the point of readmission with DKA. The patient had received the prescribed cefazolin via the outpatient parenteral antibiotic therapy service after hospital discharge. The patient also reported good compliance with prescribed medications, including oral hypoglycaemics, after hospital discharge. Nevertheless, venous blood gas results included: pH 6.87; bicarbonate 3.6 mmol/L; anion gap 30.4 mmol/L; and glucose 15.8 mmol/L. The blood ketone level was 4.3 mmol/L. The patient was treated with a variable rate intravenous insulin infusion, which was prepared by adding 50 units of Actrapid human soluble insulin to 49.5 mL of 0.9% sodium chloride and administered at insulin infusion rates of 0.5 - 8mL/hour. Intravenous isotonic sodium bicarbonate 1.26%, balanced crystalloid (Hartmann’s solution), and 10% dextrose with potassium chloride were also administered as per institutional protocol. Subcutaneous insulin therapy was commenced when the DKA resolved: insulin detemir (Levemir ^®^) 10 units twice daily and insulin aspart (Fiasp ^®^) 8 units three times daily with meals. Metformin 500 mg twice daily orally and linagliptin 5 mg once daily orally were recommenced 48 hours after readmission, with the metformin uptitrated to 1 g twice daily after one week.

At the point of readmission with severe DKA, and after having commenced systemic anticoagulation for the deep venous thrombosis, the patient reported vaginal bleeding and an ulcerated vulval lesion was noted on physical examination. Biopsies later confirmed the presence of a squamous cell carcinoma, which was staged as IVB disease on the basis of inguinal and pelvic sidewall lymphadenopathy and bone metastases present on staging scans. The vulval cancer is likely to have contributed to formation of the deep vein thrombosis as well as serving as a source of entry of *Streptococcus agalactiae* into the bloodstream ^7^.

## Discussion

Other than the introduction of empagliflozin 9 days prior, no cause for the severe DKA episode described in this case was apparent. SGLT2 inhibitors are recognised to increase risk of DKA. A meta-analysis of 39 randomised clinical trials highlighted that SGLT2 inhibitors double the risk of DKA in people with T2DM ^2^. This risk is higher in elderly patients and those using SGLT2 inhibitors for longer periods of time ^2^, neither of which applied in the current case. Whilst SGLT2 inhibitors substantially increase risk of DKA in patients with T1DM ^3^, further evaluation for T1DM after readmission with DKA was negative in the present case. Specifically, an islet autoantibody screen including antibodies to zinc transporter 8, islet antigen 2, and glutamic acid decarboxylase was negative and the 2-hour post-prandial urinary C-peptide to creatinine ratio was 3.51 nmol/mmol, indicative of substantial endogenous insulin secretion.

Apart from undiagnosed T1DM, other common precipitants of SGLT2 inhibitor-induced DKA include infection, surgery, prolonged fasting, alcohol intake, acute vascular events (such as acute coronary syndrome or stroke), trauma, and prolonged exercise ^8^. Of these, only infection is potentially pertinent to the DKA episode described herein. However, the patient’s infection was well controlled at the time of readmission with DKA. Thus, the occurrence of DKA within several days of co-prescription of empagliflozin and probenecid implicates interaction between these medications rather than other factors as the primary cause of the severe DKA observed in this case.

Empagliflozin reaches its primary site of action in the proximal tubule by a combination of glomerular filtration and tubular secretion by transporters including OAT3, which is expressed on the basolateral membrane of the proximal tubule ^6^. Probenecid inhibits OAT3 ^6^, with this property underlying its use alongside cefazolin in the present case. By diminishing renal cefazolin secretion, sufficient circulating cefazolin concentrations are achieved with once daily dosing.

In a pharmacokinetic study in healthy volunteers, co-administration of probenecid and empagliflozin, at the same total daily doses as those in the present case (1 g and 10 mg, respectively), resulted in a 26% increase in peak empagliflozin plasma concentrations and a 53% increase in area under the concentration-time curve ^6^. These findings were not felt to be clinically meaningful ^6^. However, the impact of this pharmacokinetic interaction between empagliflozin and probenecid has not been studied in patients with T2DM and it is unclear to what extent empagliflozin exposure would increase in the presence of additional OAT3 inhibitors alongside probenecid. In the present case, co-administration of empagliflozin and probenecid immediately preceded a presentation with DKA with no other apparent cause. Moreover, both benzylpenicillin and cefazolin, antibiotics administered to this patient while she was receiving empagliflozin, are OAT3 inhibitors and likely contributed to enhanced empagliflozin exposure ^9, 10^. Although not routinely available in clinical practice, the absence of plasma empagliflozin levels before and after administration of probenecid is a limitation of this case report. In the absence of drug levels confirming increased systemic exposure to empagliflozin, we can only associate rather than definitively implicate co-prescription of empagliflozin and probenecid in the aetiology of the severe DKA described herein.

Although a plausible hypothesis, certain lines of evidence argue against increased systemic exposure to empagliflozin alone being sufficient to cause the severe DKA described herein. Even if systemic exposure to empagliflozin increased by 53% with probenecid ^6^, this would not be expected to exceed the typical systemic exposure with the higher approved dose of empagliflozin (25 mg daily) - which does not routinely cause ketoacidosis. Although increased glucosuria is implicated in the mechanism of ketoacidosis with SGLT2 inhibitors ^8, 11^, the clinically approved doses of SGLT2 inhibitors (10 and 25 mg daily in the case of empagliflozin) were selected because they are near the plateau of the dose-response curve for glucosuria and are believed to achieve near-total inhibition of SGLT2 ^12, 13^. Thus, even with increased systemic exposure to empagliflozin due to probenecid, one would not expect a marked increase in the magnitude of glucosuria. Moreover, mice with genetic knockout of OAT3 had a diminished glucosuric response to empagliflozin despite comparable tubular secretion of the drug to wild-type controls ^5^. Although the physiology may differ between rodents and humans, it seems unlikely that increased glucosuria resulting in increased ketogenesis was the sole driver of ketoacidosis in the present case.

The severe DKA episode described herein may be better explained by the effects of SGLT2 inhibitors and probenecid on renal excretion of ketone bodies. Phlorizin (a non-selective SGLT1/SGLT2 inhibitor and the chemical structure upon which empagliflozin is based) increased renal tubular reabsorption of acetoacetate in dogs ^11, 14^. Through mechanisms reviewed in greater detail by Taylor *et al.*, phlorizin would be expected to exert a similar effect on renal tubular handling of β-hydrobutyrate ^11^. It is therefore plausible that empagliflozin decreases urinary excretion of ketone bodies, an effect which was compounded by co-administration of probenecid in the present case. Probenecid inhibits the organic anion transporters, OAT1 and OAT3 ^6, 15^. Knockout of the Oat1 gene in mice decreased urinary excretion of the ketone body 3-hydroxybutyrate by approximately 65% and dramatically elevated plasma concentrations of 3-hydroxybutyrate, suggesting that 3-hydroxybutyrate is a physiological substrate of OAT1 ^16^. The ligand specificity of OAT1 and OAT3 also appears to be overlapping ^17^. This raises the possibility that the severe DKA in this patient arose due to reduced urinary excretion of ketone bodies caused by concurrent treatment with probenecid and other inhibitors of renal organic anion transporters, including benzylpenicillin and cefazolin ^9, 10^, alongside the SGLT2 inhibitor empagliflozin. Of course, other factors implicated in ketoacidosis caused by SGLT2 inhibitors, such as increased ketogenesis as a consequence of glucosuria as well as increased glucagon secretion would have served to exacerbate the ketoacidosis ^8, 11^. 

In conclusion, we suggest that clinicians exercise caution when co-prescribing empagliflozin and probenecid, or indeed other inhibitors of proximal tubular organic anion transporters, which may increase systemic exposure to the SGLT2 inhibitor and/or diminish urinary excretion of ketone bodies resulting in ketoacidosis. A similar drug-drug interaction may be relevant to dapagliflozin, which is also a substrate of OAT3 ^18^. Ketoacidosis arising in this context may be severe. As co-prescription of SGLT2 inhibitors alongside inhibitors of proximal tubular organic anion transporters is likely to increase, investigation into the mechanisms of ketoacidosis in this context warrant further study. Studies assessing plasma levels of SGLT2 inhibitors and ketone bodies (β-hydroxybutyrate, acetoacetate, and acetone) before and after co-prescription of SGLT2 inhibitors with inhibitors of proximal tubular organic anion transporters in healthy volunteers and in people with diabetes mellitus would be informative.

## Consent

Written informed consent for publication of their clinical details was obtained from the patient. 

## Data Availability

All data underlying the results are available as part of the article and no additional source data are required. Open Science Framework: CARE checklist for ‘Case Report: Diabetic ketoacidosis after co-administration of empagliflozin and probenecid’. https://doi.org/10.17605/OSF.IO/8VJEQ Data are available under the terms of the Creative Commons Zero "No rights reserved" data waiver (CC0 1.0 Public domain dedication).
