# Supplementary material for: Case Report: Diabetic ketoacidosis after co-administration of empagliflozin and probenecid
Source: Wellcome Open Res. 2024 Mar 5;8:268. Originally published 2023 Jun 21. [Version 2] doi: 10.12688/wellcomeopenres.19148.2 (PMC11303938; doi:10.12688/wellcomeopenres.19148.2)
Supplement: Peer review history

# Reviewer report: Reviewer response for version 2

**DOI:** 10.21956/wellcomeopenres.23373.r89611

**Published:** 2024-08-06

**Authors:** Francis M Finucane

**Affiliations:** 1 Galway University Hospitals, University of Galway, Galway, Ireland

This is an excellent description of an interesting case which makes an important contribution to the literature about a challenging and still poorly understood clinical problem. The previous reviewer comments are insightful and constructive and the responses are comprehensive. The figure is very clear and helpful.

**I would have the following minor comments about the latest version of the case report:**

 I would specify the ethnicity of the patient at the start as it is always relevant in considering diabetes phenotype, especially in DKA occurring in type 2 diabetes.

 An excellent overview of the mechanistic basis for SGLT2i induced DKA was published last year by Fahy et al. (2023 ^1)^ and would be worth incorporating and referencing (PMID 37261785).

 It would be worth giving some specific examples when it is first mentioned that other drugs can inhibit OAT.

 It would be good to provide more clarity and specificity around the epidemiology of SGLT2i DKA - some papers suggesting they do more than just "doubling" the risk of DKA in patients with T2DM.

Are enough details provided of any physical examination and diagnostic tests, treatment given and outcomes?

Partly

Is the case presented with sufficient detail to be useful for other practitioners?

Yes

Is sufficient discussion included of the importance of the findings and their relevance to future understanding of disease processes, diagnosis or treatment?

Yes

Is the background of the case’s history and progression described in sufficient detail?

Yes

Reviewer Expertise:

Obesity, type 2 diabetes, ketone metabolism.

I confirm that I have read this submission and believe that I have an appropriate level of expertise to confirm that it is of an acceptable scientific standard.

## References

[1] : Sodium-Glucose Cotransporter 2 Inhibitor-induced Ketoacidosis and Non-Ketotic Acidosis: When Is There a Role for Bicarbonate?. *Ann Am Thorac Soc* .2023;20(6) : 10.1513/AnnalsATS.202209-780CC 911-916 10.1513/AnnalsATS.202209-780CC 37261785

# Reviewer report: Reviewer response for version 2

**DOI:** 10.21956/wellcomeopenres.23373.r75983

**Published:** 2024-04-05

**Authors:** Simeon I Taylor, Zhinous Shahidzadeh Yazdi

**Affiliations:** 1 University of Maryland School of Medicine, Baltimore, USA; 2 Medicine, University of Maryland School of Medicine, Baltimore, Maryland, USA

The revised version fully addresses the comments in our review and is a valuable contribution to the literature.

Are enough details provided of any physical examination and diagnostic tests, treatment given and outcomes?

Yes

Is the case presented with sufficient detail to be useful for other practitioners?

Yes

Is sufficient discussion included of the importance of the findings and their relevance to future understanding of disease processes, diagnosis or treatment?

Yes

Is the background of the case’s history and progression described in sufficient detail?

Yes

Reviewer Expertise:

I am a physician-scientist with a special interest in endocrinology and diabetes -- especially with respect to drugs to treat diabetes and obesity. Based on my work at Bristol-Myers Squibb where I participated in R&D leading to approval of dapagliflozin, I have specialized expertise related to SGLT2 inhibitors.

We confirm that we have read this submission and believe that we have an appropriate level of expertise to confirm that it is of an acceptable scientific standard.

# Reviewer report: Reviewer response for version 1

**DOI:** 10.21956/wellcomeopenres.21226.r72788

**Published:** 2024-02-16

**Authors:** Prashant Nasa

**Affiliations:** 1 Max Super Specialty Hospital Saket, New Delhi, India

Thank you for presenting this interesting case of DKA with the use of SGLT2 inhibitors, empagliflozin in this case. The case history is very well presented through a graphical abstract.

- However, the use of two coloured arrows to depict the drugs creates confusion. It would be better to use a single arrow for the start and end of the drug. 
- The conclusion is missing from the article. 
- The mechanisms described for DKA with the use of SGLT-2 inhibitors are multifactorial. I recommend using this review article to discuss this in detail [1].
-  Since the drug levels of empagliflozin were not measured in this case, the causal relationship between empagliflozin and probenecid drug interactions could not be established.
- I believe this should be included as a limitation.
- In addition, include a discussion on other possible mechanisms of DKA related to SGLT-2 inhibitors in this case.

Are enough details provided of any physical examination and diagnostic tests, treatment given and outcomes?

Yes

Is the case presented with sufficient detail to be useful for other practitioners?

Yes

Is sufficient discussion included of the importance of the findings and their relevance to future understanding of disease processes, diagnosis or treatment?

No

Is the background of the case’s history and progression described in sufficient detail?

Yes

Reviewer Expertise:

Intensive Care Medicine

I confirm that I have read this submission and believe that I have an appropriate level of expertise to confirm that it is of an acceptable scientific standard, however I have significant reservations, as outlined above.

## References

[1] : Euglycemic diabetic ketoacidosis: A missed diagnosis. *World J Diabetes* .2021;12(5) : 10.4239/wjd.v12.i5.514 514-523 10.4239/wjd.v12.i5.514 33995841 PMC8107974

# Response

**Published:** 2024-02-20

**Authors:** William Martin

**Affiliations:** University College Dublin, Ireland

Thank you for your thorough review of this case report and constructive feedback. We have responded to your comments by:

- Updating Figure 1 by removing extraneous arrows to improve its clarity;
- Discussing mechanisms of diabetic ketoacidosis in the setting of SGLT2 inhibitor therapy;
- Highlighting the lack of plasma empagliflozin levels before and after probenecid administration as a limitation of the case report;
- Expanding the conclusions of the article to highlight the potential for ketoacidosis with co-prescription of SGLT2 inhibitors and inhibitors of proximal tubular organic anion transporters, as well as making recommendations for future studies to tease out the mechanisms involved in the generation of ketoacidosis in this context.

# Reviewer report: Reviewer response for version 1

**DOI:** 10.21956/wellcomeopenres.21226.r68333

**Published:** 2023-10-13

**Authors:** Simeon I Taylor, Zhinous Shahidzadeh Yazdi

**Affiliations:** 1 University of Maryland School of Medicine, Baltimore, USA; 2 Medicine, University of Maryland School of Medicine, Baltimore, Maryland, USA

Martin et al. have provided an astute description of a thought-provoking case of ketoacidosis in a patient with type 2 diabetes treated with a multi-drug regimen including empagliflozin as well as probenecid. Because of the timing of ketoacidosis shortly after addition of probenecid to the therapeutic regimen, the authors hypothesize that a pharmacokinetic drug-drug interaction between empagliflozin and probenecid may have contributed to the pathogenesis of ketoacidosis. Although based on circumstantial evidence, this is a reasonable hypothesis. Specifically, the authors hypothesize that probenecid decreases organic anion transporter (OAT)-mediated renal excretion of empagliflozin. As discussed below, the literature contains substantial evidence that does not fully support their specific mechanistic hypothesis. It would have been helpful if the authors had provided data on plasma levels of empagliflozin before and after addition of probenecid. However, these analyses are not usually measured in routine clinical practice. In our review of the manuscript, we propose an alternative mechanism that might mediate a pharmacodynamic drug-drug interaction – specifically a possible role for organic anion transporters in mediating renal excretion of ketone bodies. The case report would be strengthened by adding a discussion of this alternative mechanism.

 SPECIFIC COMMENTS

1. Pharmacokinetic drug-drug interaction. Mach et al. (Clin Ther, 36, 280, 2014) [reference #6 in the paper by Martin et al.] reported formal drug-drug interaction studies indicating that probenecid increased exposure to empagliflozin by 53% (90% confidence interval, 46-61%). However, the patient was receiving the 10 mg/day dose of empagliflozin. Even if the exposure increased by 61%, the exposure would not be predicted to exceed the range of exposures typically observed in patients receiving the higher approved dose of empagliflozin (25 mg/day). Thus, it seems unlikely that the relatively modest increase in empagliflozin exposure does not seem to be sufficient to account for the patient’s ketoacidosis. Furthermore, the approved doses of SGLT2 inhibitors were selected because they are near the plateau of the dose-response curve for glucosuria and are believed to achieve near-total inhibition of SGLT2. Accordingly, a modest increase in drug levels would not necessarily be expected to increase the magnitude of glucosuria. It is also important to note that treatment emergent cases of ketoacidosis have been observed in SGLT2 inhibitors even in the absence of probenecid.
2. Role of glucuronidation in metabolic clearance of SGLTL2 inhibitors. Sarashina et al. (Drug Metab Pharmacokinet, 28, 213, 2013) reported that ~23% of the administered dose of empagliflozin was excreted in the urine. Macha et al. (Diabetes, Obesity and Metabolism, 16: 215, 2014) reported that mild-moderate renal impairment was associated with a modest increase (18-20%) in empagliflozin exposure while end-stage kidney disease was associated with a 48% increase in empagliflozin exposure. Thus, renal mechanisms are not believed to be the principal route of elimination/degradation for SGLT2 inhibitors. Rather, glucuronidation in the liver is believed to be the principal mechanism whereby SGLT2 inhibitors are cleared from the circulation.
3. Delivery of drug to its site of action in the proximal tubule. Fu et al (Am J Physiol Renal, 315, F386, 2018) [reference #5 in the paper by Martin et al.] reported that genetic knockout of *Oat3* in mice decreased the effect of empagliflozin (sub-maximally effective doses) upon glucosuria. When administered at a dose of 30 mg/kg, empagliflozin induced similar degrees of glucosuria in both wild type and *Oat3* -/- mice. Based on these data in mice, one would predict that an inhibitor of OAT3 would likely not affect the pharmacodynamic response to maximally effective doses of empagliflozin while possibly decreasing the efficacy of sub-maximally effective doses. This seems to be inconsistent with what Martin et al. have hypothesized (i.e., that probenecid increases the efficacy of empagliflozin). Of course, one cannot be certain that data in the mouse are predictive of human pharmacology.
4. A possible pharmacodynamic drug-drug interaction. Notwithstanding concerns related to the hypothesized mechanisms mediating the hypothesized drug-drug interaction, Martin et al. have provided thought-provoking circumstantial evidence suggesting that probenecid may indeed have contributed to the pathogenesis of this patient’s ketoacidosis. Accordingly, it is important to consider whether probenecid might have triggered other mechanisms that might have contributed to pathogenesis of ketoacidosis. Eraly et al. (J Biol Chem, 281: 5072, 2006) reported that knockout of the *Oat1* gene in mice decreased urinary excretion of 3-hydroxybutyrate by ~65% and also caused a dramatic increase in plasma 3-hydroxybutyrate levels. Because the ligand specificity of OAT1 and OAT3 are reported to be overlapping (Nigam et al., Physiol Rev, 95, 83, 2013), it is tempting to speculate that probenecid might also impair renal excretion of ketone bodies. (It is noteworthy that acetoacetate and 3-hydroxybutyrate are both organic anions and therefore reasonable candidates to be transported by organic anion transporters.) Furthermore, Cohen et al. (Am J Physiol, 184: 91, 1956) reported that phlorizin (the prototype SGLT inhibitor and also the chemical structure on which empagliflozin is based) promotes renal tubular reabsorption of acetoacetate. By analogy to phlorizin, it is plausible that empagliflozin might decrease urinary excretion of ketone bodies. We, therefore, hypothesize that probenecid and empagliflozin might synergize to increase plasma ketone body levels by decreasing renal clearance of those metabolities. Of course, empagliflozin-induced glucosuria indirectly triggers increased ketogenesis, which also contributes to the pathogenesis of SGLT2 inhibitor-induced ketoacidosis (Taylor et al, J. Clin Endocrinol Metab, 100, 2849, 2015).

Are enough details provided of any physical examination and diagnostic tests, treatment given and outcomes?

Yes

Is the case presented with sufficient detail to be useful for other practitioners?

Yes

Is sufficient discussion included of the importance of the findings and their relevance to future understanding of disease processes, diagnosis or treatment?

Yes

Is the background of the case’s history and progression described in sufficient detail?

Yes

Reviewer Expertise:

I am a physician-scientist with a special interest in endocrinology and diabetes -- especially with respect to drugs to treat diabetes and obesity. Based on my work at Bristol-Myers Squibb where I participated in R&D leading to approval of dapagliflozin, I have specialized expertise related to SGLT2 inhibitors.

We confirm that we have read this submission and believe that we have an appropriate level of expertise to confirm that it is of an acceptable scientific standard, however we have significant reservations, as outlined above.

## References

[1] : Safety, tolerability, pharmacokinetics and pharmacodynamics of single doses of empagliflozin, a sodium glucose cotransporter 2 (SGLT2) inhibitor, in healthy Japanese subjects. *Drug Metab Pharmacokinet* .2013;28(3) : 10.2133/dmpk.dmpk-12-rg-082 213-9 10.2133/dmpk.dmpk-12-rg-082 23149871

[2] : Decreased renal organic anion secretion and plasma accumulation of endogenous organic anions in OAT1 knock-out mice. *J Biol Chem* .2006;281(8) : 10.1074/jbc.M508050200 5072-83 10.1074/jbc.M508050200 16354673

[3] : The organic anion transporter (OAT) family: a systems biology perspective. *Physiol Rev* .2015;95(1) : 10.1152/physrev.00025.2013 83-123 10.1152/physrev.00025.2013 25540139 PMC4281586

[4] : Renal tubular reabsorption of acetoacetate, inorganic sulfate and inorganic phosphate in the dog as affected by glucose and phlorizin. *Am J Physiol* .1956;184(1) : 10.1152/ajplegacy.1955.184.1.91 91-6 10.1152/ajplegacy.1955.184.1.91 13283096

[5] : SGLT2 Inhibitors May Predispose to Ketoacidosis. *J Clin Endocrinol Metab* .2015;100(8) : 10.1210/jc.2015-1884 2849-52 10.1210/jc.2015-1884 26086329 PMC4525004

# Response

**Published:** 2024-02-20

**Authors:** William Martin

**Affiliations:** University College Dublin, Ireland

Thank you for your thorough review of this case report and constructive feedback. We have responded to your very insightful comments by:

- Providing more detailed discussion of the pharmacokinetic drug-drug interaction between empagliflozin and probenecid, and whether or not increased systemic exposure to empagliflozin alone would be sufficient explain the severe episode of diabetic ketoacidosis observed; and
- Proposing an alternative or complementary explanation for the episode of diabetic ketoacidosis arising due to the empagliflozin-probenecid drug-drug interaction, wherein both drugs act to diminish renal elimination of ketone bodies.

These points are highlighted in two new paragraphs in the Discussion of the case report. They are also highlighted in the Abstract of the revised case report. We believe that the case report now more comprehensively captures the potential mechanisms of diabetic ketoacidosis arising in the setting of empagliflozin and probenecid therapy, and recommendations are made for future studies in this field
